# Protocol for a systematic review and meta-analysis on the associations between shift work and sickness absence

**DOI:** 10.1186/s13643-022-02020-4

**Published:** 2022-07-16

**Authors:** Erlend Sunde, Anette Harris, Morten Birkeland Nielsen, Bjørn Bjorvatn, Stein Atle Lie, Øystein Holmelid, Øystein Vedaa, Siri Waage, Ståle Pallesen

**Affiliations:** 1 Department of Psychosocial Science, University of Bergen, Christies gt. 12, 5015 Bergen, Norway; 2 Department of Work Psychology and Physiology, National Institute of Occupational Health, Oslo, Norway; 3 Department of Global Public Health and Primary Care, University of Bergen, Bergen, Norway; 4 Norwegian Competence Center for Sleep Disorders, Haukeland University Hospital, Bergen, Norway; 5 Department of Clinical Dentistry, University of Bergen, Bergen, Norway; 6 Norwegian National Advisory Unit on Arthroplasty and Hip Fractures, Haukeland University Hospital, Bergen, Norway; 7 Department of Health Promotion, Norwegian Institute of Public Health, Bergen, Norway; 8 Optentia, The Vaal Triangle Campus of the North-West University, Vanderbijlpark, South Africa

**Keywords:** Working time, Work hour, Sick leave, Absenteeism, Presenteeism

## Abstract

**Background:**

Shift work, i.e., non-standard work hours, has been associated with both short- and long-term sickness absence. However, findings are inconsistent and inconclusive. Thus far, no comprehensive meta-analytic synthesis on the relationship between shift work and sickness absence has been published. The aims of the planned systematic review and meta-analysis are (1) to establish whether shift work is associated with sickness absence, (2) to determine if specific shift work characteristics relate to sickness absence (e.g., length and frequency of spells), and (3) to identify moderating factors affecting the relationship between shift work and sickness absence.

**Methods:**

Eligible studies will be identified using a predefined search strategy in several electronic databases (MEDLINE, Web of Science, PsychInfo, EMBASE, and ProQuest) and comprise peer-reviewed papers reporting original empirical findings on the association between shift work and sickness absence. Mainly observational studies with cross-sectional, prospective, or retrospective research design and case-control studies will be included. Risk of bias will be assessed using an adapted checklist previously employed to evaluate studies on sickness absence. To carry out the meta-analytic synthesis, a random effects meta-analysis will be conducted using the Comprehensive Meta-Analysis software. The review and meta-analysis will be reported according to the Preferred Reporting Items for Systematic Reviews and Meta-analyses (PRISMA) guidelines. Heterogeneity will be evaluated by *Cochran’s Q test* and the *I*^2^ statistics.

**Discussion:**

The review and meta-analysis will be the first to conduct a meta-analytic synthesis of the evidence on the association between exposure to shift work and sickness absence, as well as identify relevant moderators affecting the relationship between shift work and sickness absence. Aggregation of the existing evidence will improve the knowledge on the association between shift work and sickness absence. Such knowledge can be used to guide scheduling of shift work to promote work schedules that are less detrimental to health and contribute to reduced sickness absence and higher work- and leisure-time productivity.

**Systematic review registration:**

PROSPERO CRD42022301200

**Supplementary Information:**

The online version contains supplementary material available at 10.1186/s13643-022-02020-4.

## Introduction

Shift work involves working time scheduled outside standard daytime work hours (e.g., evening and night work) and is thereby a form of scheduling that meets the demands for 24-h operation and services [1]. Shift work is common, and among European workers, about 21% report being engaged in shift work [2]. Shift work is especially widespread in the healthcare sector with about 40% of the employees being engaged in such schedules [2]. Shift work has been associated with a range of adverse health effects, including cardiovascular disease [3], cancers [4, 5], diabetes [6], gastrointestinal disorders [7], sleep disturbances [8], and impaired mental health [9]. In addition, shift work represents a risk factor for accidents and injuries [10]. The mechanisms explaining the association between shift work and adverse health effects are not fully understood, but circadian misalignment and sleep disturbances are considered core etiological components [11–13]. Sickness absence, especially medically certified or long-term absences, can be considered as a proxy measure of workers’ health [14]. Given the associations between shift work and health problems, shift work has naturally been associated with sickness absence [15]. It should be noted that sickness absence is multifaceted and influenced also by several other factors such as social insurance systems, work environment, attitudes, and commitment [16]. Furthermore, sickness absence is not only an issue for the employee, but represents a substantial cost also for employers and social security systems [17]. Knowledge about how shift work impacts sickness absence thus is of major significance, both from the employees, the employers, and the societal perspective.

Shift work and sickness absence are both multifold and complex phenomena. As for shift work, it is common to distinguish between permanent shift work schedules (e.g., permanent night work), rotating shift work schedules (e.g., regular/fixed three-shift rotation: alternating between morning, evening, and night shifts), and roster work schedules (irregular rotation between shifts) [18]. In addition, shift work can be described according to several other dimensions such as direction and speed of rotation, duration of shifts, length of shift cycle, and regularity of schedules [19]. In a similar manner, sickness absence can be quantified using different measures including frequency of sickness absence, length of absence, and duration of spells/episodes [16]. It is also common to distinguish between short- and long-term sickness absence, as the two aspects of sickness absence may be explained by different factors. Ill health is considered a strong predictor of long-term sickness absence while psychosocial factors, in addition to ill health, may be more important for short-term sickness absence [20]. Still, definitions of short- and long-term sickness absence vary and are inconsistent. For instance, in a recent study from Norway, short and long periods of sickness absence were defined as 1–8 days and ≥ 9 days, respectively [21]. The aforementioned distinction was used as the studied population of hospital employees had requirements for a medical certificate only for sickness absence of ≥ 9 days. However, a study from Finland defined short sickness absence as 1–3 days [22], whereas another study among Danish and Finnish nurses defined long-term sickness absence as ≥ 30 consecutive days off [23]. Taken together, shift work and sickness absence are not uniform and precise concepts and the definitions vary across studies. The planned review and meta-analysis will be broad scoped and aspire to include any variations and definitions of the constructs. Applicable distinctions between categories of the constructs will be applied in analyses.

The status of the current knowledge base on shift work and sickness absence is, to some extent, likely to be a result of the extensive variation in the theoretical and operational definitions of the constructs. A previous systematic review on the association between shift work and sickness absence concluded that findings were inconsistent and inconclusive [15]. However, a positive association between fixed evening work and sickness absence among female nurses was reported; hence, the association between shift work and sickness absence may be schedule and population specific [15]. The notions of inconsistency and schedule-specific associations seem to be valid also for recent studies. For instance, one study found that quick returns (< 11 h of rest between shifts) were associated with a higher risk of sickness absence, while night work was not [24]. Another study reported that working mainly night shifts was associated with short-term sickness absence among full-time retail workers [25]. One study indicated that two-shift rotation (days and evenings), three-shift rotation (days, evenings, and nights), and fixed night work were related to elevated short-term sickness absence, but findings were inconclusive for long-term sickness absence [21]. Others have found higher risk of long-term sickness absence with both consecutive night shifts and evening work [23]. However, it has also been reported that having a high number (> 25%) of evening shifts and ≥ 2 consecutive evening shifts are associated with lower risk for sickness absence [22]. As findings appear to be inconsistent across studies, there is a need to systematically and quantitatively summarize and update the evidence on the association between shift work and sickness absence.

There are several potential moderators of the relationship between shift work and sickness absence. These include demographic variables such as age, gender, and having children [21], in addition to differences across countries [23]. Other relevant factors comprise health and lifestyle (e.g., physical activity, alcohol consumption, smoking status) factors, as well as work-related factors (e.g., position, tenure, part- and/or full-time employment, psychosocial and physical work environment) [15, 26]. Many of the latter factors can also be considered as confounding variables. Some recent studies have adjusted for confounders, e.g., weekly work hours and length of shift [21, 25], but the previous systematic review noted that many studies did not adjust for such variables [15].

In order to provide new knowledge on how shift work impacts sickness absence, the planned study will provide a systematic review and a meta-analytic synthesis of the available research literature. In reference to Participants, Intervention, Comparators, and Outcomes (PICO), participants will here comprise workers, the intervention consists of exposure to shift work/non-standard work hours, the comparator reflects day work/non-shift work, and the outcomes are all types of sickness absence/sick leave indicators. The main aim is to determine whether exposure to shift work, i.e., non-standard work hours, is related to sickness absence, either short or long term. The meta-analysis will establish the overall magnitude of this association. Furthermore, as the association between shift work and sickness absence may be schedule specific, it is an aim to establish the magnitude of the association for different shift schedules. For the purpose of the planned review and meta-analysis, shift work schedules will generally be categorized as follows: permanent shift work schedule (i.e., permanent/fixed early morning, evening, or night work), rotating shift work schedule (i.e., regular/fixed shift rotation with night work, regular/fixed shift rotation without night work), and roster work schedule (i.e., irregular shift rotation with night work, irregular shift rotation without night work). Roster work includes schedules commonly seen among healthcare workers with irregular two- or three-shift rotation. In addition, quick returns (< 11 h of rest between shifts) will be considered as a form of shift work. Depending on the identified studies, long work hours (i.e., ≥ 9-h and/or ≥12-h shifts) and/or the number of consecutive evening or night shifts will also be included as separate shift work categories. Sickness absence will be categorized, as either short or long term, in order to assess if the relationship between shift work and sickness absence differs for short- and long-term sickness absence. Initially, long-term sickness absence will be defined as ≥ 30 consecutive days off, but the precise definition may however be adjusted depending on the definitions employed in the identified primary studies.

The role of moderating factors affecting the relationship between shift work and sickness absence will also be investigated. Based on the literature, age, gender, and occupation/profession, as well as geographical context (i.e., living/working country), seem to be relevant moderators. Studies have often included many different confounding/adjustment variables in addition to age and gender; hence, the number of adjustment variables will also be assessed in meta-analyses.

## Methods

This protocol has been written based on the PRISMA-P (Preferred Reporting Items for Systematic Review and Meta-Analysis Protocols) guidelines and the MOOSE Guidelines for Meta-Analyses and Systematic Reviews of Observational Studies [27, 28]. In the Additional file 1, a completed PRISMA-P checklist is provided.

The planned review and meta-analysis is part of a larger project entitled “Towards a sustainable work force in the healthcare sector for the 21^st^ century: Health-promoting Work Schedules” (HeWoS). The overarching aim of the project is to establish a knowledge base on health-promoting work schedules among healthcare workers and to facilitate a productive and sustainable work force in the healthcare sector in the future.

### Information sources

To collect data for the literature review and meta-analysis, systematic searches in multiple electronic databases will be conducted, including MEDLINE, Web of Science, PsychInfo, EMBASE, and ProQuest. Additional searches will be conducted in Google Scholar, and reference lists of key articles will be assessed to identify any eligible studies. Data from other sources, i.e., conference abstracts, dissertations, gray literature, and unpublished data, will not be included. The initial literature search was conducted in December 2021. An update search will be conducted during autumn 2022. The main search will be performed by a professional librarian at the Norwegian Institute of Public Health.

### Search strategy

A specific search strategy will be developed for each database based on combinations of two categories of search terms (i.e., shift work and sickness absence). The searches will be adjusted according to the requirements for each specific database (i.e., use of operators and symbols). An example of the search strategy and search terms is included in Table 1. The searches will be limited to peer-reviewed articles written in Danish, Dutch, English, French, German, Italian, Norwegian, Spanish, or Swedish. Historical time constraints will not be put as a limitation for the searches. The risk of selection and detection bias is considered to be sufficiently reduced using this search strategy. While a professional librarian conducts the main search, the primary investigator will oversee the searches. The search results will be exported to Endnote, where duplicates will be removed. The results will subsequently be exported to the Rayyan software [29].

**Table 1:** Search strategy developed for the MEDLINE database

1	MeSH terms: Shift work schedule/ or Work schedule tolerance/ or "Personnel staffing and scheduling"/ or Workload/
2	(("personnel staffing" adj2 scheduling) or "work schedule?" or workschedule? or ((shift or shiftwork* or "shift work*") adj (schedule? or system? or pattern? or arrangement? or work* or plan* or roster? or rota?)) or "work table?" or worktable? or ((night or overnight or evening or afternoon or "after noon" or "early morning?" or rotat* or rotary or irregular or "non standard" or nonstandard) adj1 (work* or shift? or shiftwork* or "shift work*" or schedule?)) or nightshift? or nightwork* or overnightwork* or overnightshift? or (work* adj (time? or hour? or week*)) or worktime? or workhour? or workweek* or "split shift?" or "swing shift?" or "long shift?" or "extended work hour?" or "extended workhour?" or "roster work*" or "short rest" or "quick return?" or ((employee or staff) adj ("work load?" or workload?))).tw,kf.
3	1 or 2
4	MeSH terms: Sick leave/ or Absenteeism/ or Presenteeism/
5	(((disability or sick* or medical or injur* or absen*) adj (leave? or day? or absen* or spell or episode? or list*)) or "day off" or (work adj3 (absen* or "day loss" or "time loss")) or Presenteeism or presenteism or "sickness presence" or ("working while" adj (ill or sick))).tw,kf.
6	4 or 5
7	3 and 6
8	Occupational health/ or ((employee or occupational) adj health).tw,kf.
9	7 and 8
10	7 or 9
11	limit 10 to "reviews (maximizes specificity)"
12	Meta-Analysis/ or Network Meta-Analysis/ or ((systematic* adj2 review*) or metaanal* or "meta anal*" or (review and ((structured or database* or systematic*) adj2 search*)) or "integrative review*" or (evidence adj2 review*)).tw,kf,bt.
13	11 or (10 and 12)
14	10 not 13
15	limit 14 to (danish or dutch or english or french or german or interlingua or italian or multilingual or norwegian or spanish or swedish)

### Eligibility criteria (inclusion and exclusion criteria)

Eligible studies report original empirical findings on the association between exposure to shift work (any concept of non-standard work hours) and sickness absence (e.g., self-certified/self-declared, certified by medical doctor, short and/or long term, registry-based sickness absence). Included studies will be quantitative observational studies with cross-sectional, prospective, or retrospective research design; case-control studies; and studies with experimental designs. Studies with qualitative designs and single-case studies will be excluded.

The study population will be workers (≥ 15 years of age) with current or previous conditions of employment. No restrictions will be put on participants regarding gender, ethnicity, or other demographic variables. As one of the main aims is to investigate moderating factors, age and gender will be used as moderators in meta-analyses. Provided that a sufficient number of relevant studies are available, occupation/profession (e.g., healthcare [nurse vs. medical doctor], industry, transportation, police and emergency, etc.) and living/working country will also be included. Moderator analyses will either be performed as subgroup analysis or meta-regression. To perform a basic meta-analysis, a minimum of two studies is required [30].

The magnitude and direction of associations between shift work and sickness absence will be determined using cross-sectional and prospective data, respectively. For inclusion in the meta-analysis, studies must provide the zero-order associations (e.g., correlations, odds ratios [OR]) between shift work and sickness absence or provide sufficient information for such effect sizes to be calculated. For studies lacking such information, authors will be asked to provide data. If the necessary information cannot be obtained, the study will be excluded from the meta-analyses. Double-counting of data will be avoided by ensuring that the sample in a given study do not overlap with samples included in any previous and already included studies. If overlap occurs, data from the largest sample will be used.

### Data management and selection process

Two independent reviewers, ES and AH, will screen the articles identified in the searches based on the criteria provided in Table 2. The Rayyan software will be used to organize the screening and review process. At the first level of screening, the search results will be considered based on the title and abstract. At the second level, the full-text articles passing the first screening and articles with insufficient information will be evaluated in terms of effect sizes and methodology. If opinions differ, the reviewers will discuss the matter to reach consensus. If necessary, a third reviewer will be consulted. This procedure minimizes bias when deciding to include or exclude studies.

**Table 2:** Selection table for inclusion or exclusion

Language and literature	Design	Exposure	Control group/comparator	Outcome	Data analysis	Results
Peer-reviewed, full-text, European language	Observational study: cross-sectional, prospective or retrospective, case-control, cohort, experimental	Shift work: non-standard work hours	Non-shift work/day work: working hours between 06:00h and 18:00h	Sickness absence/sick leave	Analyses with comparison between shift work and control groups	Results of comparison between shift work and the control group (or provided by authors)

### Data extraction

Data will be extracted using a predefined data extraction/coding form by the two reviewers, independently. A coding form will be developed and pre-tested on relevant articles, and then adjusted if needed, before being employed to all the included studies. Training and discussions about how to code variables according to the form will be conducted in order to ensure high interrater reliability. This is expected to improve precision and contribute towards reviewers conducting congruent coding. The coding form will register information about shift work and sickness absence, demographic information, study characteristics, and other relevant variables. The following data will be extracted and coded: first author name and publication year, living/working country of sample, data collection period, study design, sample of shift workers (nurses, police, industry, etc.), sampling method, response rate, sample size (total, female, male), age of participants (range, mean [*SD*]), shift work type (early morning, evening, night, rotating, permanent, etc.), other included work characteristics (tenure, length of shift, weekly work hours, etc.), assessment method (registry, self-report, etc.), length of sickness absence (short or long term), moderators and confounding variables, shift work, and sickness absence effect sizes (correlations, OR, coefficients, etc.): both unadjusted/minimum adjusted (adjusted/unadjusted = 1/0) and fully adjusted (n adjustment variables). If the reviewers’ extractions differ, discussion and further review of the studies will be conducted until consensus is reached.

### Assessment of risk of bias

The two reviewers will assess the risk of bias of the included studies using a 14-item checklist (Table 3), similar to a checklist used in a previous meta-analysis addressing sickness absence [31]. The checklist is adapted from developed tools for assessing risk of bias in prevalence studies [32] and non-randomized studies [33]. The checklist will be tested on relevant articles, and a manual will be developed to ensure that the reviewers employ similar criteria when evaluating the studies. The overall quality of each study will be scored on a scale from 0 (lowest quality) to 14 (highest quality). The total risk of bias scores will be categorized as follows: high risk (0 to 5), moderate risk (6 to 10), and low risk (11 to 14). To assess and quantify interrater agreement, Cohen’s kappa will be calculated.

**Table 3:** Checklist for the assessment of methodological quality of the reviewed studies

	Points
**Sampling and representativeness**	
1. Sampling method	
A. Nonprobability sampling (including purposive, quota, convenience and snowball sampling)	0
B. Probability sampling (including simple random, systematic, stratified g, cluster, two-stage and multi-stage sampling) or registry (i.e., full sample/all workers)	1
2. Was the response rate reported?	
A. Not reported	0
B. Response rate below 50%	0
C. Response rate at 50% or above	1
3. Are the individuals selected to participate in the study likely to be representative of the target population?	
A. No	0
B. Yes	1
4. Selection bias: Is there a risk of selection bias caused by the inadequate selection of participants?	
A. High risk	0
B. Low risk	1
5. Is the sample size adequate for establishing relationships (assumption of statistical power)?	
A. No	0
B. Yes	1
**Measurement and confounders**	
6. How was working time assessed?	
A. Self-report	0
B. Objective data (including registries and payroll records)	1
7. How was sickness absence assessed?	
A. Self-report	0
B. Objective data (including registries and payroll records)	1
8. Performance bias: Is there a risk of bias caused by the inadequate measurement of exposure?	
A. High risk	0
B. Low risk	1
9. Are the statistical methods appropriate for the study design?	
A. No/Can’t tell	0
B. Yes	1
10. Were meaningful demographic covariates included?	
A. No	0
B. Yes	1
11. Were other work factors adjusted for?	
A. No	0
B. Yes	1
12. Is the study design cross-sectional or prospective (with time-lag)?	
A. Cross-sectional	0
B. Prospective	1
13. Was previous sickness absence adjusted for in prospective analyses?	
A. No	0
B. Yes	1
14. Confounder bias: Is there a risk of bias caused by the inadequate confirmation and consideration of confounding variable?	
A. High risk	0
B. Low risk	1

### Meta-analysis

To conduct the meta-analysis, the Comprehensive Meta-Analysis version 3 (Biostat Inc., USA) will be used. As an overall measure of effect size, OR with 95% confidence intervals (CI) will be reported. In a first step, a general meta-analysis on shift work and sickness absence will be conducted. If studies report several effect sizes from the same sample (e.g., studies presenting models with different control measures), the mean of the combined effect sizes will be calculated. For studies with overlapping samples, the study with the largest number of respondents will be included. We expect that several studies will report effect sizes from independent subgroups separately. For instance, independent effect sizes may be reported for those engaged in night work and those not engaged in night work, or when moderators (e.g., male and female, different age groups) are included. In such circumstances, each subgroup will be included in the meta-analysis as a unique sample, provided that participants/groups are not double counted. In a second step, separate analyses for the different shift schedules (e.g., permanent night work, regular shift rotation with and without night work) will be conducted, as well as for sickness absence type (i.e., short-term and long-term sickness absence) provided that meaningful distinctions can be made.

Moderator analysis will be conducted in an attempt to assess and account for differences in the relationship between shift work and sickness absence. Provided that the number of studies is sufficient (i.e., ≥ 2), age, gender, occupation/profession, and country will be assessed by comparing effect sizes for subgroups. In addition, many studies have included different confounding/adjustment variables in addition to age and gender. Thus, the number of adjustment variables will be included as a moderator in a meta-regression. The main meta-analyses will use effect sizes from unadjusted (minimum adjusted) models, then the effect of adjustment variables will be assessed in a meta-regression. Relevant methodological moderators will also be analyzed to address whether aspects such as study design and measurement methods impact findings. For instance, how sickness absence has been assessed in the primary studies (e.g., number of days, number of spells).

Studies will be weighted by the inverse of the variance, which is roughly proportional to the sample size [34] and which is based on the DerSimonian and Laird approach [35]. Pooled mean effect sizes will be calculated using a random effects model, as the different studies are assumed to reflect different population of studies. To assess the heterogeneity of the studies, *Cochran’s Q test* will be used, with a significant *Q*-value rejecting the null hypothesis of homogeneity. To indicate the relative heterogeneity (the proportion of variance which reflect true variance between studies), the *I*^2^ statistic will be calculated, with 25% indicating low heterogeneity, 50% indicating medium heterogeneity, and 75% indicating high heterogeneity, respectively [36]. To identify whether overall estimates are influenced by outlier studies, a “one-study-removed” approach will be used. Outlier studies will be those falling outside the 95% CI for the average effect size. The funnel plot [37], Orwin’s Fail-Safe N (trivial OR set to 1.2 and 1.5) [38], Duval and Tweedie’s trim and fill procedure [39], and Eggers regression intercept [37] will be examined as indicators of publication bias and strength/stability of associations [34].

## Discussion

The review and meta-analysis will systematically summarize the evidence on the association between exposure to shift work and sickness absence. Information about moderating factors affecting the relationship will also be collected, and the findings will extend the knowledge base and understanding of how shift work affects sickness absence. This knowledge can potentially guide employers to implement shift work schedules that are less detrimental to health and hence reduce or minimize sickness absence and improve work- and leisure-time productivity.

There are several potential limitations with the review and meta-analysis that should be noted. It will be based on data from peer-reviewed studies only; hence, unpublished studies and non-peer-reviewed literature will not be included. Thus, potential sources of information may be missed. Likewise, as only European languages will be included, information in other languages will be missed. Including only peer-reviewed studies may improve the quality of the data, but issues concerning risk of “publication bias” will not be reduced [40]. It is likely that studies will be heterogenous in terms of, e.g., the populations studied, but also in terms of definitions of shift work and sickness absence. This may limit comparability and interpretation of the results. The meta-analysis will include studies with cross-sectional designs and the aggregated effect sizes will therefore not account for the causal relationship between the included variables. Separate analyses will be conducted for studies based on time-lagged data in order to determine the direction of associations over time.

## Supplementary Information

**Additional file 1.** PRISMA-P 2015 Checklist.

## Data Availability

Not applicable.

## Abbreviations

PRISMA: Preferred Reporting Items for Systematic Reviews and Meta-analyses
OR: Odds ratio
SD: Standard deviation
CI: Confidence intervals
